# Contextual factors experienced by mothers of children with special needs

**DOI:** 10.4102/sajp.v80i1.2060

**Published:** 2024-10-25

**Authors:** Priscilla Matambanadzo, Anthea J. Rhoda

**Affiliations:** 1 Department of Child and Family Studies, Faculty of Community and Health Sciences, University of the Western Cape, Bellville, South Africa; 2 Department of Physiotherapy, Faculty of Community and Health Sciences, University of the Western Cape, Bellville, South Africa

**Keywords:** caregivers, special needs, children, contextual factors, international classification of functioning disability and health

## Abstract

**Background:**

Caring for children with special needs places a burden on caregivers. The challenges experienced can be conceptualised as personal and environmental, as per the International Classification of Functioning, Disability and Health Framework (ICF).

**Objectives:**

Supported the caregiver experiences when caring for children with special needs and to conceptualise these needs in relation to the personal and environmental factors set out in the ICF.

**Method:**

A qualitative exploratory study was conducted, using purposive and snowball sampling. Semi-structured interviews were used to collect the data, which were then analysed thematically.

**Results:**

A total of six themes arose from the data. Two of these were related to personal factors: unemployment and caregiving, and marital uncertainty. The other four themes were related to environmental factors: family and caregiving factors, educational factors, health-systems challenges and perceptions of children with special needs.

**Conclusion:**

Caregivers of children with special needs experienced personal challenges, which resulted in feelings of isolation from family and society. They also faced environmental challenges in sectors such as education and health.

**Clinical implications:**

The management of children with special needs should include interventions within their environments to assist caregivers with their tasks. This could contribute to improved quality of life for both the caregivers and their children.

## Introduction

Caregivers of children with disabilities experience a number of challenges that include social isolation (Currie & Szabo 2020) and are often family members of the children, usually their mothers or fathers. The caring process has an impact on caregivers, including stress that negatively affects their health (Masefield et al. 2020). The family is often psychologically impacted (Coetzee 2016). There is also a greater financial burden on the family, which contributes to increased stress on the family as well as the carers (Geiger 2012). Providing care for children with special needs has a significant impact on caregivers, negatively affecting their mental, physical and financial well-being. Individuals frequently encounter mental turmoil, displayed as sensations of powerlessness and remorse (Coetzee 2016; Masefield et al. 2020), while persistent stress can give rise to physical ailments (Geiger 2012; Masefield et al. 2020). Financial strain stems from the additional costs linked to medical treatment and support services, leading to economic difficulties (Geiger 2012; Masefield et al. 2020).

Children with disabilities have special needs and they rely almost entirely on other individuals for their basic care (Olusanya et al. 2023). A child with special needs is therefore greatly dependent on others. The care needed by these children includes psychosocial support (such as emotional encouragement and social interaction facilitation, to address psychological challenges), feeding and assistance with mobility (including assisting the child with walking, transferring or transitioning to different positions or using mobility aids such as wheelchairs or walkers) (Geiger 2012).

In South Africa, the majority of people with disabilities, including children with special needs, are excluded from mainstream society (Bingham 2017). Families with children with special needs are also often marginalised (Bingham 2017). Although socioeconomic challenges affect most of the South African population, the situation is even worse for children with special needs and their parents or caregivers. Parents or caregivers face challenges because of the scarcity of resources such as respite care and have difficulty in finding people capable of providing constant supervision for children with special needs (Geiger 2012). Parents of these children are also isolated by their extended families, which results in avoidance of attending family and community events (Zuurmond et al. 2019). Being part of a support group, which counteracts feelings of isolation, is viewed positively by caregivers of children with special needs (Kalman, 2016). It must, however, be noticed that the experiences of families and caregivers of children with special needs might vary (Kalman 2016).

The personal and environmental experiences of caregivers caring for children with special needs may be conceptualised in terms of the International Classification of Functioning, Disability and Health Framework (ICF) (World Health Organisation [WHO] 2001). According to the ICF, personal factors include those related to the individual’s sex, race, education, fitness, lifestyle, habits and coping styles. Environmental factors relate to the physical, social and attitudinal milieu within which people function. Environmental factors can be described at an individual level and a societal level. At the individual level, environmental factors include the individual’s immediate environment, such as the home, school and workplace; at the societal level, they include structures, social networks and systems that influence and impact the lives of individuals (WHO 2001).

The experiences of caregivers will differ depending on the context of the caregiver. The aim of our study is to present the personal and environmental factors experienced by caregivers of children with special needs. Information from our study could be used to inform the implementation of strategies to assist this group of individuals, which would impact not only the children but also on the mothers and the broader family. The study, in addition, has responded to the need to expand the body of knowledge in the field of human functioning and disability (Threats 2007).

## Research methods and design

Mfuleni is one of the rural communities located approximately 32 km from the central area of Cape Town, South Africa. The township with a population of approximately 52 300 (Statistics South Africa 2011) is primarily inhabited by individuals of African descent, although there are some individuals of mixed-race heritage living in this community as well. The majority of individuals were relocated to the Mfuleni area during the late 1990s as a result of flooding and fires in various townships across the Western Cape, including Philippi, Nyanga and Khayelitsha. Unemployment, HIV/AIDS and high levels of crime are among the most urgent issues impacting this impoverished township.

A qualitative exploratory design (Babbie et al. 2001) was used to collect the data in our study. The data were collected by conducting semi-structured interviews based on an interview guide that assisted in structuring these interviews. The interview guide was developed through a systematic process by P.M. to ensure the thoroughness and relevance of the qualitative enquiry. A thorough literature review was performed to identify the themes and matters of relevance to children with special needs and their caregivers to guide the development of the interview guideline.

### Sampling strategy

Purposive and snowball sampling was used to recruit participants for the study. To qualify for selection, they had to be a caregiver of a child with special needs, with the latter defined as a child needing any form of assistance with the basic activities of daily living as a result of cognitive impairment and/or physical disability. Participants were recruited via a community-based organisation catering for children with special needs. Saturation was achieved when eight individuals were interviewed and no new information emerged, after which no further recruitment took place.

### Data collection procedure

Once the ethics clearance and necessary permission from the institutional committee were obtained, the researcher approached the social worker of the community-based organisation and requested permission to access the organisation’s database in order to source patient information, within the guidelines of the *Protection of Personal Information Act* (PoPIA). In addition to meeting with the social worker, the researcher met with the manager of the community-based organisation as well.

Thereafter, the researcher approached the selected participants telephonically or in person and, with the assistance of a community development worker, invited them to be part of the study. The recruitment process took 2 to 3 weeks (from 02 October 2017 to 21 October 2017). Once participants had been recruited, they were invited for an interview at a place and time suitable for them. All the interviews were conducted at a local library because it was a quiet, safe and central space for participants within the vicinity of their homes.

The researcher informed the participants about the research process, including its recruitment phase, and explained that confidentiality and anonymity would be maintained during and after the data collection. The latter process commenced once written informed consent was obtained from the participants. The consent included permission to audio-record the interviews. The interviews conducted by the researcher were audio-recorded, were 30 min – 45 min in duration and were conducted in two local languages, namely isiXhosa and Afrikaans. The participants were asked about their perceptions, challenges and coping strategies as caregivers caring for a child with special needs. In addition to asking questions based on the interview guide, the researcher documented demographic information about the participants, including their age, sex, employment status and relationship with the child. After the interviews, language experts were used to translate the transcripts that were conducted in isiXhosa and English. A research assistant who acted as an interpreter assisted with the data collection.

### Data analysis

Thematic analysis, based on the guidelines by Creswell (2007), was used to methodically explore and examine the patterns of significance within the interview data. Firstly, interviews performed in languages other than English were translated by language experts into English to maintain uniformity in the analysis. The interviews were recorded and transcribed word for word to correctly reflect the subtleties of participants’ responses. Secondly, the transcribed data underwent a comprehensive review and coding process to discover re-occurring themes and categories. The coding technique entailed methodically assigning labels to text segments that encapsulated crucial concepts or ideas pertaining to the study enquiry. P.M. was primarily involved in the data analysis with input from A.J.R. Through a systematic and repetitive examination of codes, the authors were able to identify and categorise overarching themes that represent the primary experiences and viewpoints of caregivers who look after children with special needs. By employing a thematic analysis approach, a thorough investigation of the data was conducted, enabling an in-depth understanding of the difficulties, encounters and requirements of caregivers in the specific context of tending to children with special needs.

### Trustworthiness and rigour of the study

The study ensured rigour and trustworthiness by adhering to the principles of conformability, credibility, dependability, transferability and a reflexive approach to the enquiry and analysis (Shenton 2004). The study’s confirmability was ensured by presenting exact transcripts of the participants’ responses. The study’s credibility was upheld by granting participants the opportunity to freely articulate their thoughts during the interviews. Furthermore, to ensure freedom of expression, member verification or checking was performed at the conclusion of each interview. This process involved summarising the main concepts discussed throughout the interview.

In order to ensure reliability, the procedures used for collecting and analysing data were thoroughly described. Moreover, the research team utilised an interview guide to direct all of the interviews. P.M. conducted the interviews with the assistance of a research assistant. The researchers discussed and verified the data collection procedures and analysis. To ensure transferability for our study, a comprehensive methodology was presented, which encompassed the study setting, participants and data collection processes. The study team maintained a reflective notebook throughout the research procedure. A record comprising the responses, exchanges, considerations and resolutions was created by the study team members during the research endeavour. This included the audio recordings and the written notes documented in the reflective journal.

### Ethical considerations

Approval for the study was obtained from the Human and Social Sciences Ethics Committee of the University of the Western Cape (HS/16/7/11). Permission to conduct the study was obtained from a community-based organisation in the Western Cape, South Africa. Written informed consent was obtained from the participants. Respect for the individuals was maintained as the participants were informed that their participation in our research is completely voluntary and that they had the right to withdraw at any time without providing any reasons. Participants were notified that their true identity will be concealed by using pseudonyms during the process of analysing and discussing the data. This was implemented to facilitate the anonymity of the data and to ensure that information could not be linked to any specific individual. Furthermore, confidentiality was ensured as all the data collected throughout the investigation were securely stored on a password-protected computer which only the research team could access.

## Findings and discussion

### Sample

A total of eight participants were recruited to be part of the study. All participants interviewed were mothers of the child with special needs. Cerebral palsy, autism spectrum disorder and traumatic brain injury were the diagnoses of the children with special needs whose caregivers were interviewed. Caregiver participant ages ranged between 28 and 39 years. All the interviewed participants were unemployed females, living in a rural community in South Africa and were mothers of the children.

### Personal factors

Two themes related to personal factors emerged. They were unemployment and caregiving, and marital uncertainty.

### Unemployment and caregiving

The inability of caregivers to seek employment as a result of having to care for their children with special needs often increases the financial burden on them. Although all participants indicated that they received a disability grant to the value of ZAR 1600 per month, it was not enough to enable them to provide for the needs of their children. For many of them, this grant is the only source of income as they are unemployed. Zuurmond et al. (2019) also concurred that caring for a child with a disability creates a financial burden for individuals as they often have to relinquish employment in order to do so.

One of the participants said that, while she appreciated the grant, it was not sufficient for meeting the needs of the child:

‘It is really not enough even for the child, as they need clothing nappies, special foods, and transport when attending doctors’ appointments.’ (Female, 36 years old, unemployed)

Women (mothers) in these situations are very often disadvantaged economically because although they have the desire to seek employment, work and obtain an income, they cannot do so because of their circumstances. A lot of time is required to take care of a child with special needs.

### Marital uncertainty

The fear of being left abandoned with a child with a disability is real. Once the diagnosis of the disability becomes known, this could lead to marriages breaking up and spouses being deserted (Duma, Tshabalala & Mji 2021). As a participant observed:

‘The father of my child left me the first time I told him that the child has a disability.’ (Female, age 38, unemployed)

Fathers struggle to accept the fact that their child has a disability. Another participant, referring to her spouse, said:

‘He told me he never [*fathered*] a disabled child.’ (Female, 28 years old, unemployed)

There is hence a need to educate fathers of children with disabilities, especially about the fact that their contribution to the care of the child would decrease the burden on the mother (Cramm & Nieboer 2011). The lack of knowledge of alternative means of enforcing care from the father results in fathers being either absent or playing only a part-time role in caring for the child.

However, even caregivers who receive financial support from their husbands’ experience fear and anxiety of being deserted by them, as is illustrated by the following quote:

‘I also think of the future. What if my husband leaves us? How are we going to survive with R1 600 with my kids? These the things that frustrate me more [*sic*].’ (Female, 32 years old, unemployed)

Maternal caregivers’ fear of desertion appears to stem in part from their direct reliance on the financial contribution of the father figure in the household, as he is the one who is available for employment. Where the father figure is absent, however, the financial challenges facing caregivers are aggravated when they (the caregivers) have no information about alternative recourse (Ambikile & Outwater 2012). Primary caregivers should therefore be educated about the possibility of seeking legal recourse to ensure that they are assisted in meeting the costs of caring for children with special needs (Oti-Boadi 2017).

As mothers are expected to become caregivers of children, which could affect their economic activity, the sex differences in their caregiving role could be questioned. Although the mother and father may have differing roles, this should not imply that the woman should bear the sole responsibility for attending to the child’s daily needs (Ndadzungira 2016). Similarly, a father’s duty to contribute as a caregiver is not automatically nullified if he chooses to leave without making any contribution to the child’s sustenance and daily care. As indicated in the given quote, the mothers expressed a level of frustration and not being able to cope should they be left alone caring for their child with special needs. Family members of the father also contributed to these feelings of the mothers as:

‘Even now his mother does not even bother supporting him [*husband*] with this child though she is staying here in Cape Town.’ (Female, 36 years old, unemployed)

In view of these various factors, female caregivers find themselves in a position where they could lose the support of their counterparts. The relationship between the mother and father of the child disintegrates to some extent because of the amount of time that the mother spends caring for the child. For instance, one of the study participants was forced to move to Cape Town from the Eastern Cape to obtain better medical care for the child. Her relocation meant she had to leave the father of her child behind:

‘It extremely affected my relationship because I am here in Cape Town alone and he is in the Eastern Cape, as I had to move for better treatment and medications in the Western Cape. We are still having a relationship, but it is difficult.’ (Female, 36 years old, unemployed)

### Environmental factors

Environmental factors impacted the study participants on both an individual level and a societal level and took the form primarily of barriers. Barriers at the individual level related to family and caregiving; societal environmental barriers related to challenges in accessing education and healthcare and to disability stereotyping in communities.

#### Individual-level environmental barriers

One theme emerged related to individual-level environmental barriers, namely, family and caregiving.

**Family and caregiving:** Children with special needs may be subjected to stigma and rejected by their family because of their disabilities. Such stigmatisation could be directed towards the family as well. In this regard, the following quote illustrates how a participant experienced stigmatisation by members of her family:

‘At first his family [*husband*] did not want him to involve himself with his child as they said that in their family there are no disabled children. They were influencing him not to meet his child… [*they also said*] that I had another man that I was sleeping with because their brother and family cannot have disabled children.’ (Female, 28 years old, unemployed)

The perceptions and attitudes towards children with disabilities in South Africa are challenging, as highlighted by Oti-Boadi (2017). Societal attitudes and preconceptions can lead to misunderstandings and prejudice, whereas poor communication impedes effective comprehension. Moreover, the presence of various viewpoints among caregivers, educators and healthcare professionals makes the process of understanding even more complex. Oti-Boadi (2017) highlighted the significance of addressing these challenges by adopting a comprehensive and compassionate strategy that takes into account cultural attitudes, communication obstacles, varied viewpoints and environmental elements to foster inclusivity and assistance for children with disabilities. The participants in this study experienced accusations of promiscuity against them. Emotional support by the families (including their husbands) of children with disabilities could assist caregivers to relate better to their children emotionally and interact with them more positively. Also, such support will positively affect their mental health. Furthermore, family support is important because it helps caregivers to accept their children and find effective ways of caring for them (Ndadzungira 2016). Conversely, a lack of family support may lead to caregivers rejecting their children or blaming themselves for the child’s disability or questioning what they did to deserve a child with special needs. This is reflected in the following quote:

‘I could not even look at my child that time. I asked myself, what did I do to deserve this?’ (Female, 38 years old, unemployed)

Caregivers often appeared to engage in some degree of self-blame, as they believed that it was their fault that the child has special needs. Statements such as the one above show that an immediate reaction to having a child with special needs may be to see the situation as a curse or a form of punishment (Meadan, Stoner & Angell 2010).

Another participant opined that:

‘It took time for my family to accept that I have a disabled child.’ (Female, 33 years old, unemployed)

Her siblings would pass comments such as:

‘When are you going to put your cripple down?’ (Female, 33 years old, unemployed)

These remarks show that it is often hard for a family to support a caregiver with a child with special needs. The language used by family members when communicating with caregivers about their children indicates stigmatisation. Research has shown that siblings and fathers of children with special needs may experience a lack of knowledge and sympathy regarding their needs (Giallo et al. 2013; Gray 2006). Siblings may experience feelings of being disregarded or not fully understood within the family dynamic, while fathers may grapple with feelings of inadequacy or ambiguity regarding how to effectively support their child with special needs. In addition, caregivers often find themselves isolated from friends and relatives because of the demands and course of events associated with taking care of a child with special needs (Currie & Szabo 2020; Oti-Boadi 2017).

Caregivers’ emotional state can be adversely affected as they try to manage the socially perceived cause of the child’s disability, performing their expected caregiving roles while coping with everyday livelihood challenges in order to provide for the child.

#### Societal-level environmental barriers

McNally and Mannan (2013) observed that a lack of finances for necessities, including healthcare, often hinders caregivers’ access to required services. However, in some instances, as observed by Taderera and Hall (2017), the available public resources themselves may not be accommodating to children with special needs, for instance, education institutions, modes of transport or other public infrastructure meant to be accessible to everyone.

In this regard, the study’s second objective was to explore the participants’ experiences of access to services (healthcare, education, social care). Participants in the study indicated that they faced numerous challenges in accessing healthcare and education.

### Difficulties in accessing the education system

There is a limited number of schools that cater for children with special needs in South Africa and mothers have difficulty in finding such schools close to where they live (Donohue & Bornman 2014). A participant stated that:

‘You cannot move, there are no schools that can accommodate my child for me to look for employment.’ (Female, 32 years old, unemployed)

Furthermore, children with disabilities may have challenges with the mainstream education system. A participant highlighted that she had to remove her child from attending one such institution:

‘I did take her out of crèche because she was not coping.’ (Female, 39 years old, unemployed)

Her comment shows that a child with special needs have difficulties or challenges coping within the schooling environment; the reasons for this are myriad. This study identified accessibility and accommodation challenges, safety concerns, diversity of needs, financial constraints, limited availability of special schools and struggles within mainstream education as significant barriers faced by children with special needs in the schooling environment. These barriers have also been identified in previous studies (Donohue & Bornman 2014; Grant & Jones-Goods 2016) and have limited participation as a result of challenges with access and availability of resources (Africa, Human & Tshabalala 2023).

Teachers and staff at schools and creches may not have the skills to accommodate children with special needs (Grant & Jones-Goods 2016). One participant reported:

‘Sometimes she [*her child*] gets sick because they do not adjust her sitting position.’ (Female, 28 years old, unemployed)

Another participant suggested that schools that cater for children with special needs should separate the children in accordance with their disability. She stated:

‘In a school for children with autism, they should separate the children, because the child with autism is dangerous to my child because mine cannot even move an arm.’ (Female, 28 years old, unemployed)

Her concern highlights that schools should consider the specific disability and determine the type of learning environment, which is best suited to the child.

Finding schools that are inclusive of children with special needs, especially in the areas where the participants reside, has proven to be difficult, as illustrated in the following quotes:

‘I am still on the waiting list at […]. I do not know when they will call to confirm that she got a place or not. This is the most challenging thing for me, because other children do go to school and she cannot [*sic*].’ (Female, 30 years old, unemployed)

‘I also want her to go to school but I hardly can get a school. The schools here in [*in the local community*] are not accommodating [*reference to a specific area*] children because there is no transport to [*this area*].’ (Female, 38 years old, unemployed)

### Difficulties in accessing healthcare services

Study participants reported that the lack of follow-up communication from healthcare professionals was among the reasons that it was difficult for them to accept, and cope with, the disability of their children. This is illustrated in the following quotation:

‘[*T*]he physiotherapist took my number, promising to call me for another appointment, but until today I have not received any call.’ (Female, 28 years old, unemployed)

Caregivers also have challenges keeping appointments at healthcare facilities as a result of a lack of funds:

‘The income for the household is very small and her disability grant is not enough, as I have to hire transport to take us to appointments.’ (Female, 30 years old, unemployed)

The lack of information from healthcare professionals can exacerbate the difficulties caregivers face in accepting and managing their child’s disabilities. When promised appointments or follow-up sessions fail to materialise, caregivers might also feel neglected or deserted by the healthcare system, increasing their feelings of isolation and frustration. This loss of communication undermines caregivers’ acceptance of the healthcare system and also hinders their ability to continue accessing available support for their child (Mwangi et al. 2022).

Moreover, financial constraints pose enormous obstacles to access healthcare facilities and services. Caregivers regularly have difficulty finding the means to pay for transport to attend clinic or hospital appointments, especially while their family income is small and the disability grant obtained for the child is insufficient to cover those expenses. As a result, caregivers can be pressured to prioritise vital needs over gaining access to healthcare, doubtlessly compromising the continuity and effectiveness of their child’s physiotherapy and other essential interventions (Mwangi et al. 2022).

### Perceptions of children with special needs

Perceptions of children with special needs are influenced by belief systems and the information people are exposed to about disability. Communities tend to stigmatise children with special needs, a phenomenon which can be linked directly to failure to understand disability (Hearst et al. 2020). In turn, most (maternal) caregivers of children with disabilities are isolated as a result of this lack of knowledge in the wider population and the association that various belief systems make between disability and the supposed infidelity of the mother or being cursed (Oti-Boadi 2017). Various cultures hold strong ideas and prejudices about disability, such as connections to supernatural origins, punishment for previous wrongdoings or bringing disgrace to the family. These perceptions contribute to the stigmatisation and isolation of mothers who are caretakers for individuals with disabilities (Mwangi et al. 2022). These cultural perceptions can result in unjust accusations or exclusion among communities. Moreover, the societal expectations and duties associated with roles might intensify the strain on maternal caregivers, resulting in heightened feelings of seclusion and exclusion from social circles. Comprehending the cultural context is crucial in dealing with the intricate interaction of elements that impact the experiences of mothers caring for children with disabilities (Mwangi et al. 2022) and designing support treatments that are culturally appropriate.

In this regard, participants reported experiences in which negative statements were made about their children. For example:

‘They will call your child names like “cripple” [*isidalwa*], and people will comment that they do not have a cripple in their family.’ (Female, 28 years old, unemployed)

‘Sometimes people will ask why the child does not walk and will pass remarks when I carry her on my back, saying she is too old, why can’t she walk? Do they think you can just carry a big, heavy child on your back for no reason?.’ (Female, 33 years old, unemployed)

Stigmatisation, name-calling and stares can all contribute to adverse effects on the caregivers’ state of mind; caregivers can therefore present with depression and other mental health conditions. Stigmatisation affects not only the child but all with whom they are associated. Caregivers therefore experience ‘courtesy stigma’ as a result of their position as the parents and primary caretakers of children with special needs (Maldonado, Martins & Ronzani 2023).

The community should refrain from stigmatising children with disabilities and rather seek to understand the nature of the disability and offer communitarian help. This would not only be in line with South Africa’s constitutional values of ubuntu (a concept deeply rooted in African-origin value systems. It emphasises the interconnectedness of individuals with their surrounding societal and physical worlds) but also create an environment where the caregiver and the child with special needs do not feel neglected and isolated.

### Clinical implications

Based on the findings presented in the research, there are some potential clinical implications.

Firstly, a need for psychosocial support services: The study emphasises the considerable emotional and psychological difficulties experienced by mothers of children with disabilities. This highlights the necessity of providing easily available psychological therapy and support services to assist caregivers in managing stress, isolation, self-blame and marital strain.

Secondly, family counselling and education: These are crucial in addressing the stigma, a lack of acceptance and insensitive comments that family members direct towards a child with a disability. Providing education to extended family members about disability could promote the development of a more supportive atmosphere.

Furthermore, a paternal involvement programme is recommended: Findings from the study indicated that the mothers perceived that fathers frequently face difficulties in accepting their child’s impairment and may relinquish their responsibilities as caregivers. Initiatives focussed on enhancing paternal engagement should consider including the fathers.

Caregiver training and respite care: Caregivers expressed challenges in effectively placing and tending to their children’s unique requirements. Implementing focussed training programmes that teach specific caring skills, in addition to providing easy access to respite care facilities, has the potential to improve the quality of care.

Community awareness campaigns: Public awareness programmes are necessary to address the prevalent stigma and lack of understanding surrounding impairments in communities. These initiatives have the potential to question and debunk prejudices, foster inclusivity and enhance societal backing for individuals who provide care.

Improving accessibility of services: The challenges faced in obtaining suitable education, healthcare and transportation emphasise the necessity for more inclusive public services and improved connections to resources for children with disabilities.

Lastly, financial and legal support: Considering the economic disadvantage and concerns regarding child support, caregivers may find it helpful to receive advice on financial aid programmes and where possible approach legal aid services to facilitate financial contributions from the child’s father.

### Limitations

The lack of recruitment of fathers into the study is a key limitation. Fathers could have provided a perspective different to that of mothers and one based on a different experience. Moreover, the fact that all the mothers were recruited via a single organisation is also a limitation, in that the children had similar levels of disability. A sample from a broader population could have expanded the findings. The adoption of a qualitative methodology means that although the design of our study can be replicated, the findings it yields are idiosyncratic to its specific research setting and therefore limit the generalisability of the findings. The lack of information about the functional levels of the children which has an impact on caregiving is another limitation of the study. Additionally, a lack of information related to the demographics of the mothers, such as their environmental contexts and circumstances, is another limitation of the study.

## Conclusion

Our study highlights the complex difficulties experienced by mothers caring for children with special needs, encompassing both personal and environmental levels. Personal factors such as being unemployed and having uncertainty in one’s marriage have a substantial impact on caregivers’ ability to meet their children’s needs. At the same time, environmental factors, such as challenges in obtaining healthcare and education services, worsen the strain of caregiving. The results emphasise the pivotal significance of dynamics in caring obligations for mothers, emphasising the necessity for healthcare practitioners to promote increased participation of fathers in assisting children with special needs. Moreover, it is crucial to prioritise the resolution of societal stigma and discrimination towards children with disabilities and their families to cultivate an inclusive and supportive community environment. In the future, efforts should prioritise offering extensive assistance to caregivers, advocating for equitable representation in caregiving responsibilities and increasing knowledge to combat prejudices and foster acceptance of children with special needs. By confronting these difficulties and cultivating a nurturing atmosphere, we can improve the welfare and quality of life for both caregivers and their children with special needs.
